# Regular and random judgements are not two sides of the same coin: Both representativeness and encoding play a role in randomness perception

**DOI:** 10.3758/s13423-021-01934-9

**Published:** 2021-05-06

**Authors:** Giorgio Gronchi, Steven A. Sloman

**Affiliations:** 1 Department of Neuroscience, Psychology, Drug Research and Child’s Health (NEUROFARBA) – Psychology Section, University of Florence, via di San Salvi n. 12, Complesso di S. Salvi, Padiglione 26, 50139 Florence, Italy; 2 Cognitive, Linguistic and Psychological Sciences, Brown University, Providence, RI USA

**Keywords:** Randomness perception, Representativeness, Encoding, Reaction times

## Abstract

The overalternating bias is that people rate sequences with an excess of alternation as more random than prescribed by information theory. There are two main explanations: the representativeness heuristic (Kahneman & Tversky *Cognitive Psychology, 3,* 430–454, 1972) and the implicit encoding hypothesis (Falk & Konold *Psychological Review, 104,* 301–318, 1997). These hypotheses are associated with different reaction times predictions. According to the encoding hypothesis, reaction times should increase as the complexity of the sequence increases, whereas the representativeness heuristic predicts fast reaction times only for more complex sequences that appear more random. We asked participants to guess the generating source of pairs of sequences of dichotomous elements in two different conditions: selecting the string generated by a random source or selecting the string generated by a nonrandom source. Results suggest that both the encoding strategy and the representativeness heuristic have a role in randomness perception and that the two criteria may have a different weight when determining the randomness versus the regularity of a string.

**Supplementary Information:**

The online version contains supplementary material available at 10.3758/s13423-021-01934-9.

## Introduction

Decisions made in the face of sequential data generally require that we establish whether two or more events are related in some way (e.g., that they have a common generating source), or that they are unrelated and any perceived connection between them is merely a coincidence. Many real-life examples testify to people’s tendency to see patterns and regularities in outcomes generated by a random process: from the belief of British citizens of London that there were clusters of strikes during World War II bombings, to iPod users’ claim about the nonrandomness of the (actually random) shuffle function (Froelich et al., 2009; Levy, 2005). On the one hand, seeing a pattern where none exists can bring people to ask themselves for an explanation about the origin of a nonexistent relationship, potentially leading, for example, to superstitious beliefs. On the other hand, failing to detect an existing regularity means missing some hidden generating rule, a rule that in scientific and other contexts can be important.

A lot of effort has been devoted to understand randomness perception (see reviews by Nickerson, 2002, 2004; Oskarsson et al., 2009). A recurring result is that stimuli with more alternations are often identified as most random (Falk, 1975, 1981; Falk & Konold, 1997; Gilovich et al., 1985) violating normative criteria derived from information theory (see Attneave, 1959; Gronchi et al., 2016). For example, strings such as XXXOXXOOXOO (maximally random according to information theory) are rated less random than OXXOXXOXOOX (less random according to a normative criterion but with more alternations). This overalternating bias represents a robust and consistent finding in human randomness perception (Falk & Konold, 1997).

Kahneman and Tversky (1972) proposed the use of the representativeness heuristic (a similarity judgement between an observed event and the mental prototype of randomness) to explain the intuitive notion of randomness. A string of dichotomous elements is judged random (or regular) if it is similar to its parent population (the family of random sequences or the family of regular sequences) and reflects the salient features of the process by which it was generated (a random process or a regular process). For random sequences, the main traits of their population (i.e., an equal number of both elements and an irregular order) must be present in a string to be considered random. This leads to local representativeness: the idea that these two characteristics (equiprobability and irregularity) should manifest in each short segment of a string generated by a random process. It is natural to conclude that building a sequence using these criteria will create a sequence with too much alternation compared with the features of a typical random string. With regard to a regular process, the logic is the same: A sequence drawn from a regular population should represent some kind of pattern or law.

Falk and Konold (1997) claimed that randomness judgments are based on an (almost) implicit attempt to encode the string (or, equivalently, an implicit assessment of the sequence’s difficulty of encoding). If the sequence is hard to chunk (i.e., an implicit attempt to encode it or to judge its complexity), the string will be judged random. On the contrary, if the sequence is easily chunked, the sequence is considered not random. To measure difficulty, Falk and Konold (1997) propose the difficulty predictor (DP): the number of runs (i.e., substrings consisting of only tails or heads) plus twice the number of alternating substrings. Higher scores indicate a harder to chunk sequence. For example, XXXXXXXX is a single run of *X*s, thus DP = 1 whereas XXXXOOOO and XOXOXOXO both have DP = 2 since the first string is composed of two runs of a single element and the second is a single run of alternating elements).

The DP measure is strongly correlated with randomness ratings of strings and with various measures of its encoding difficulty (actual hardness of memorization, assessed difficulty of memorization, copying difficulty, task of segmentation). Falk and Konold’s (1997) interpretation is that randomness may be equated with the difficulty of encoding a sequence. Their hypothesis is that observers attempt to chunk the sequence and use the difficulty or the outcome of the attempt to decide the randomness of the sequence. Falk and Konold do not compare their hypothesis with the representativeness criterion. Yet, despite the procedural difference between the encoding criterion and the representativeness heuristic, both strategies lead to the same predictions about randomness ratings. Indeed, hard to chunk sequences are the strings highly representative of a random process and Falk and Konold’s data are also compatible with local representativeness. Falk and Konold’s (1997) work shares a key assumption of the randomness perception literature: Evaluating possible regularities in a sequence gives information about its perceived randomness and vice versa. As Kubovy and Gilden (1991) wrote “apparent disorder and randomness might be expected to be the converse of apparent regularity, meaningfulness and redundancy. That is, one might think that the less regularity people find in a sequence, the more likely they are to consider it random” (p. 116).

The interchangeability between random and regular judgements is supported by research that asked subjects to judge matrices made up of green and blue dots and manipulated the instructions (Zhao et al., 2014): In one condition, participants were asked to identify the half of a display more likely to be produced by a random process. In the other condition, they were asked to identify the half of the display more likely to be produced by a nonrandom process. The authors did not find an effect of the framing of the question on identification performance.

However, in tasks asking subjects to assign the generating source (random or nonrandom) of sequences of heads and tails, we observed correlational data suggesting a difference between random and nonrandom responses (Gronchi & Sloman, 2009): faster reaction times for random responses and reaction times proportional to the complexity of the sequence for regular responses. Here, we explore the possibility that different kinds of instructions (asking to detect a random string as opposed to asking to detect a regular string) affect judgements about the generating source. We hypothesize that reaction times might distinguish the two questions even when accuracy is comparable. In particular, if the task is to decide if a sequence is regular, we expect response times to increase proportionally with sequences’ complexity (in line with the prediction of the encoding model). In contrast, if the task is to decide if the sequence is random, we expect fast and complexity-independent reaction times (a pattern that is compatible with a similarity judgement to a prototypical random pattern).

## Method

### Participants

Forty students of the University of Florence (26 females) with a mean age 21.6 years *(SD* = 2.5) volunteered. None were expert on probability or statistics.

### Stimuli and procedure

Participants were presented with two eight-character sequences (composed of *X*s and *O*s). We employed all possible sequences composed of eight characters. There are 256 of them, but each configuration of elements has two equivalent forms (e.g., XXXXXXXX is equivalent to OOOOOOOO), so we only tested half (128 sequences). The whole set of strings was divided into three groups using the DP measure^1^: low subjective randomness (DP between 1 and 2), intermediate subjective randomness (DP between 3 and 4) and high subjective randomness (DP between 5 and 6).

Each participant was randomly assigned to one of two conditions: nonrandom or random. In the nonrandom condition, the observer had to choose the most likely two strings to be produced by a regular process, whereas in the random condition, the observer had to choose the most likely of the two to be produced by a random process. There were six possible types of pairs: low/low complexity, intermediate/intermediate complexity, high/high complexity, low/intermediate complexity, low/high complexity and intermediate/high complexity.

For each trial, the software showed two sequences (displayed in a random order on the screen). The strings were selected according to the following rules: (i) all the possible different pairs of low/low subjective randomness (36 pairs) sequences; (ii) 36 pairs of high/high subjective randomness sequences (chosen randomly from the pool of high subjective randomness sequences); (iii) 81 low/high complexity sequences (each one of the possible nine low complexity strings paired with other nine high complexity sequences randomly selected from their pool; (iv) 200 pairs selected with the same probability from the remaining possible pairs (intermediate/intermediate complexity, low/intermediate complexity, and intermediate/high complexity) with each sequence randomly selected from the respective pool of sequences and the constraint of not generating already showed pairs. In total, each participant observed 353 different pairs of sequences.

Participants assigned to the Nonrandom condition read this text:

You are going to see sequences composed of *X*s and *O*s that have been generated either by a random process where both characters have the same probability of occurrence (such as tossing a fair coin with an *X* on one side and an *O* on the other side) or by another kind of nonrandom process (in other words, a process that chooses the characters on the basis of a defined rule). You are going to see two sequences at once, one produced by the random process and the other by the regular process. Your task is to decide as fast as possible which one has been produced by the regular process. Remember that your speed will be measured.

After you click on “Start the task,” place your right hand on the keyboard with your index finger on the *O* key and the middle finger on *P* key, whereas your thumb will be positioned on the space bar. To see a new pair of sequence you will press the space bar; once you see the pairs of sequence to judge you will press *O* or *P* to indicate which sequence has been produced by a regular process.

O = left sequence

P = right sequence

In the random condition, the description of the task was: “Your task is to decide as fast as possible which one has been produced by the random process” and, at the end of the text, the participants were asked to press *O* or *P* to indicate which sequence has been produced by a random process. The experiment was carried out individually in a quiet setting among other nonrelated computer-administered experiments.

### Data analysis

Reaction times ranged from 252 to 14,892 ms. Short reaction times were long enough to allow the observer to make a decision, and thus no entry was deleted. A log_10_ transformation was applied to the reaction time data in order to better approximate a Gaussian distribution. An a priori power analysis was performed before carrying out the experiment. Data were analyzed by means of a 2 × 6 repeated-measures ANOVA, with log_10_ reaction times as a dependent variable, condition (nonrandom source vs. random source) as a between variable, and complexity (low/low complexity, intermediate/intermediate complexity, high/high complexity, low/intermediate complexity, low/high complexity and intermediate/high complexity) as the within variable.

## Results

An a priori power analysis was conducted using G*Power3 (Faul et al., 2007) with a small effect size (*f* = .3), and an alpha of .05. It showed that a total sample of 40 with two equal sized groups of *n* = 20 was required to achieve a power of .80.

When asked to select the string generated by a random process between low and intermediate complexity strings, participants chose the latter sequence in 85.3% of cases. In the case of the low/high comparison, high complexity strings were chosen in 89.5% of trials and in the case of intermediate/high comparison, the high complexity sequences were chosen in 68.4 of cases.

When asked to select the string generated by a regular process between a low and intermediate complexity string, participants chose the low complexity sequence in 83.5% of trials. In the case of the low/high comparison, low complexity sequences were chosen in 88.1% of cases and in the case of intermediate/high comparison, the intermediate complexity strings were chosen in 73.6 of cases.

When both strings had the same complexity, the selection between the left and the right sequence were nearly at chance. The percentages selecting the leftmost sequence were 44.4%, 43.9%, 43.5% for random condition low/low complexity, intermediate/intermediate complexity and high/high complexity, respectively. When asked to select the sequence generated by a regular process, the percentages selecting the leftmost sequence were 57.6%, 49.4%, 46.5%, for low/low complexity, intermediate/intermediate complexity, and high/high complexity, respectively.

**Fig. 1:**
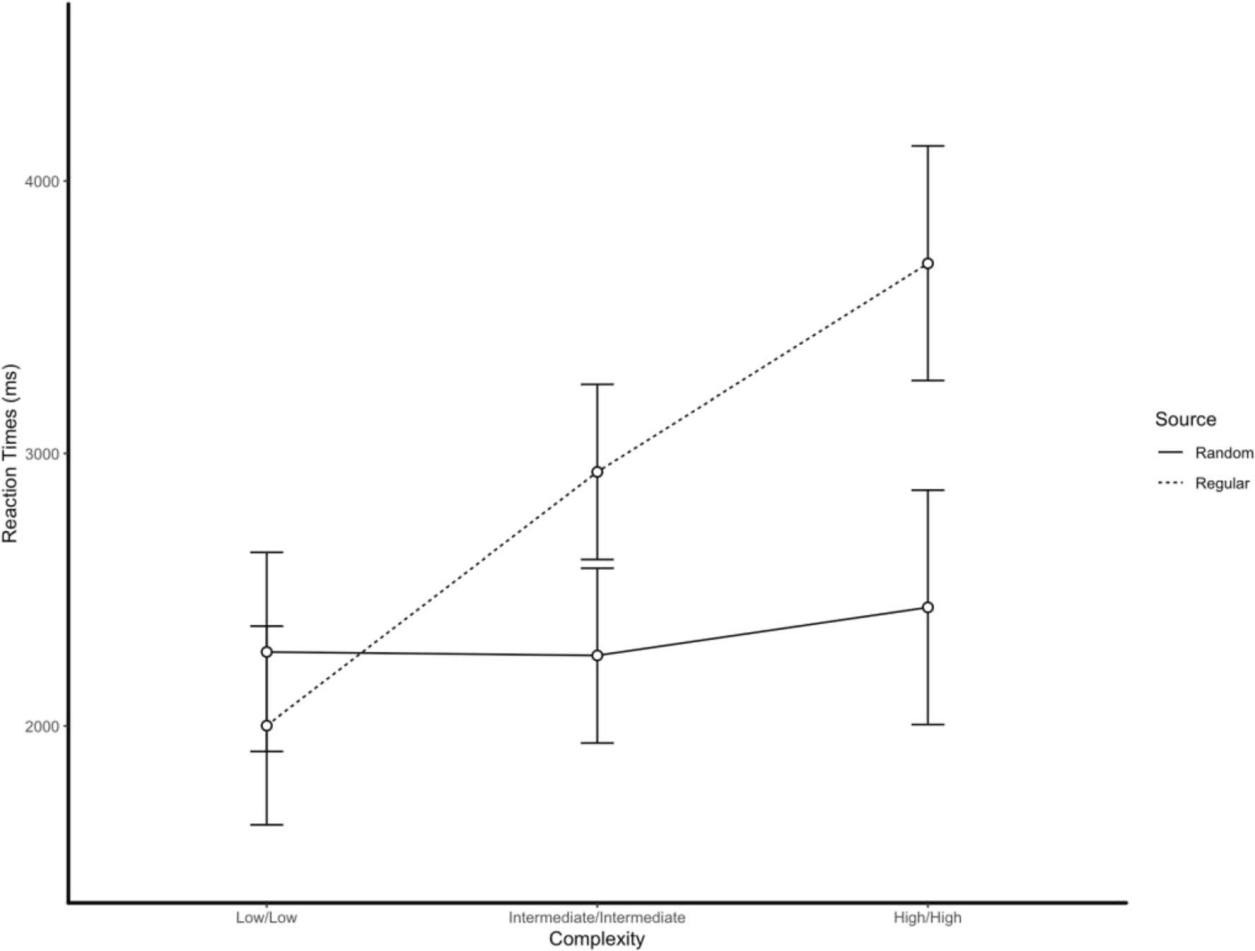
Reaction times as function of the complexity (low/low, intermediate/intermediate, and high/high) of pairs of sequences and condition (selecting the sequence generated by the random source vs. selecting the sequence generated by the nonrandom source). When asked to select which of the two sequences was generated by the random source, reaction times were fast and independent of complexity. When asked to select which of the two sequences was generated by the nonrandom source, reaction times were proportionally to the complexity. Error bars represent 95% confidence intervals

**Fig. 2:**
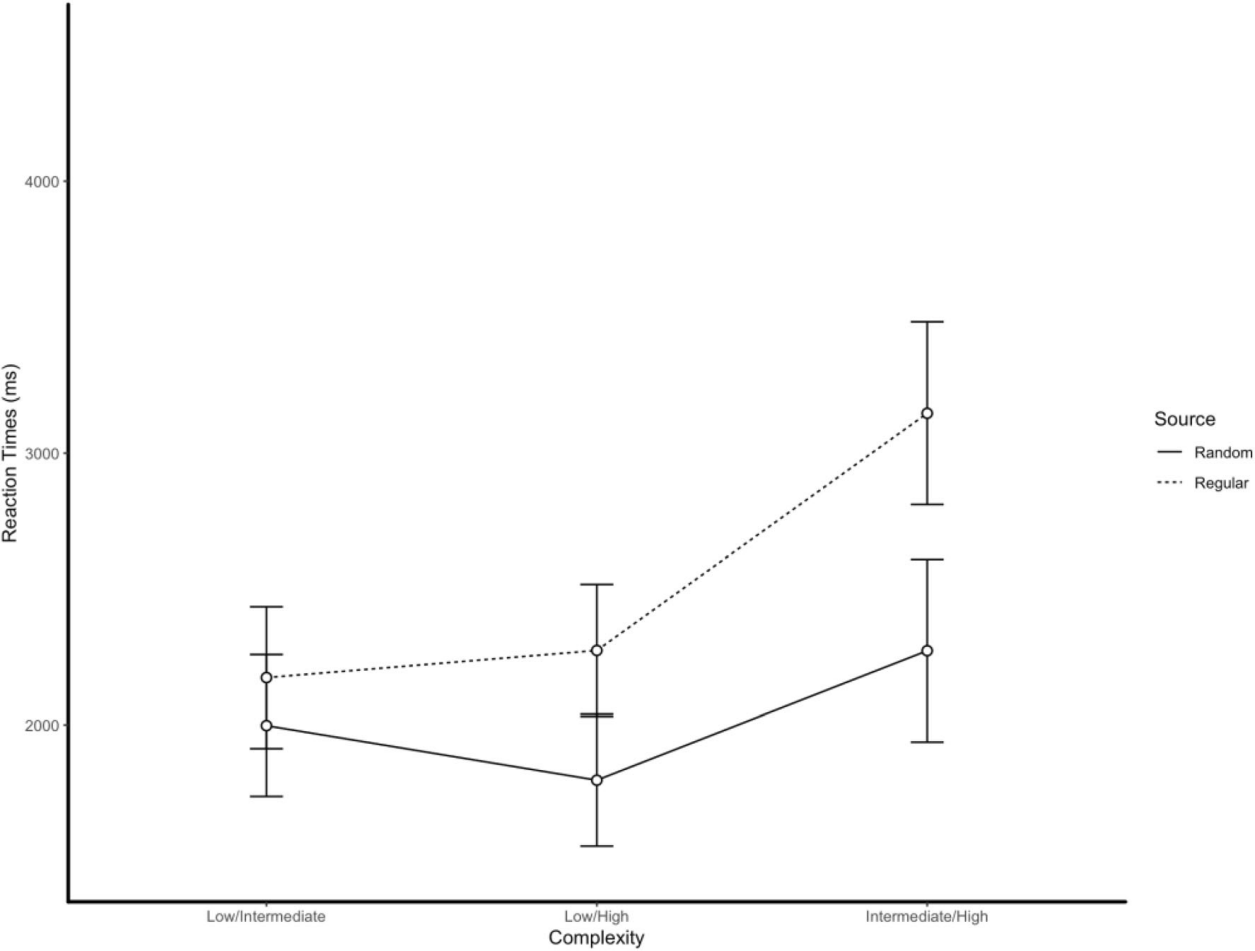
Reaction times as function of the complexity (low/intermediate, low/high, and intermediate/high) of pairs of sequences and condition (selecting the sequence generated by the random source vs. selecting the sequence generated by the nonrandom source). When asked to select which of the two sequences was generated by the random source, reaction times were fast and independent of the kind of comparison. When asked to select which of the two sequences was generated by the nonrandom source, reaction times were significantly higher in the intermediate/high condition. Error bars represent 95% confidence intervals

Reaction times showed significant main effects of complexity, *F*(5, 190) = 45.28, *p* < .001, and condition, *F*(1, 38) = 5.90, *p* = .020, and the Complexity × Condition interaction was statistically significant, *F*(5, 190) = 18.10, *p* < .001 (see Figs. 1 and 2).^2^

**Fig. 3:**
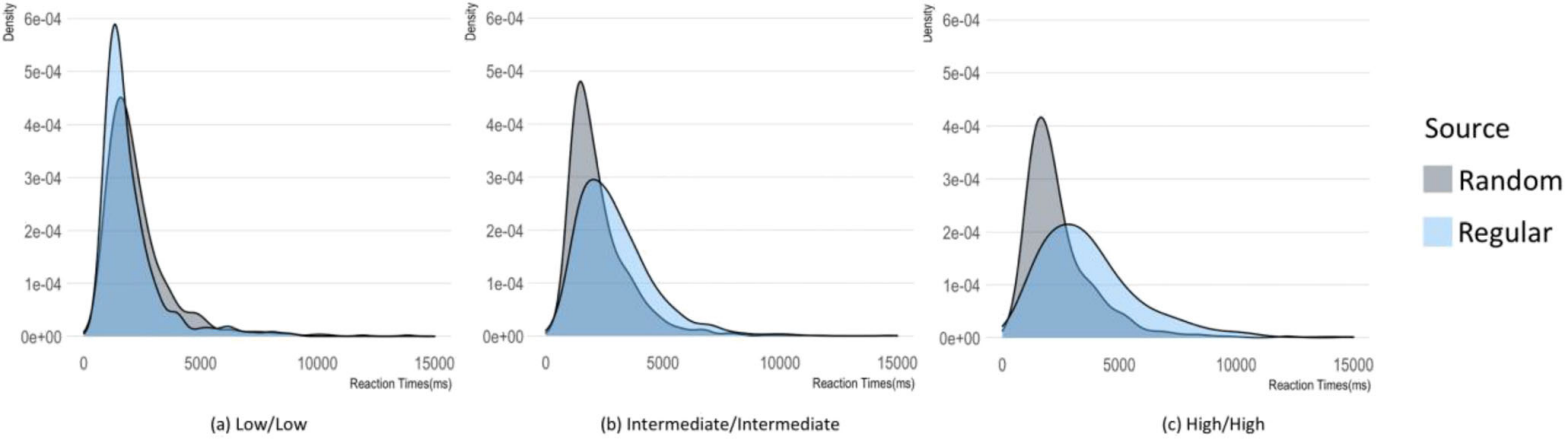
Density distribution of reaction times as a function of the complexity (low/low, intermediate/intermediate, and high/high) of pairs of sequences and condition (selecting the sequence generated by the random source vs. selecting the sequence generated by the nonrandom source). RT distributions in the random condition were similar across complexity, whereas the RT distributions for regular responses have a progressively lower peak as the complexity increases

**Fig. 4:**
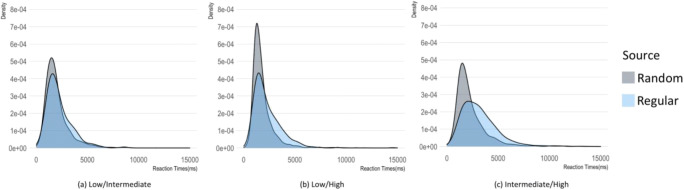
Density distribution of reaction times as a function of the complexity (low/intermediate, low/high, and intermediate/high) of pairs of sequences and condition (selecting the sequence generated by the random source vs selecting the sequence generated by the nonrandom source). RT distributions in the random condition were similar for low/intermediate and intermediate/high, with a faster front-end peak for the low/high pairs of string. RT distributions in the regular condition were similar for low/intermediate and low/high complexity with a longer tail for intermediate/high condition

The analysis of reaction times distributions (Figs. 3 and 4) confirmed these results. With regard to judgments of same-complexity pairs, the distributions in the random condition were similar across complexity, whereas in the regular condition the peak tends to decrease as the complexity increases. When judging pairs of sequences with different complexity, the distributions for Random responses were similar for low/intermediate and intermediate/high, with a higher and earlier peak for the low/high pairs of sequences. Reaction times distributions in the Regular condition were similar for low/intermediate and low/high complexity whereas in the intermediate/high condition we observed a lower peak with a long tail.

## Discussion

Random and regular judgments are usually seen as two sides of the same coin in the randomness perception literature: Evaluating the randomness of the sequence automatically gives information about its regularity and vice versa. Indeed, a shared (and often implicit) assumption of the randomness perception literature is that the less regularity one is able to detect in a stimulus, the more likely the observer tends to perceive it as random (Falk & Konold, 1997; Kubovy & Gilden, 1991).^3^ The consequence of this assumption is the conception that a common (and commonly assumed to be unique) process determines both the evaluation of randomness and regularities.

Our result challenges this idea: We observed a different pattern of reaction times when people had to choose the most random of two sequences compared with the most regular. The observed response times are compatible with the idea that people use both a similarity judgement for evaluating the randomness of a sequence and the encoding strategy for evaluating its regularity. However, this interpretation requires some caveats. The representativeness heuristic and the encoding strategy models have been developed assuming an observer that is judging a single sequence and they have been expressed in very general terms. In order to make more specific predictions about reaction times, the process that leads to decision requires a more detailed description.

As far as we know, Diener and Thompson (1985) is the only study that has tried to analyze reaction times in order to compare two specific hypotheses about randomness perception. They contrasted the representativeness strategy with another theoretical model, randomness-by-default. They assumed that greater representativeness (either in terms of similarity toward the prototype of a random string or a regular string) would lead to lower reaction times: Highly representative sequences will be classified more quickly than those not representative of either. According to randomness-by-default, the observer starts to look for any possible regularities: If all attempts fail, the observer responds “random”. Randomness is not actively searched for in the sequence; it is a default judgment if the attempt to find regularities fails.

They observed that, when judging 20-element sequences, reaction times for “yes” responses to the question “is the coin fair” were longer than for “no” responses and the latter increased linearly with confidence ratings about the strength of belief that each sequence was produced randomly. We did not find this, perhaps because of a difference in task demands (judging a single sequence compared with choosing the most random between two). Nevertheless, Diener and Thompson’s (1985) data are compatible with our hypothesis on the assumption that an encoding strategy is deployed to respond to the question, “Is the coin fair?”—that is, that this question is treated as one about regularity. The two findings might also differ because Diener and Thompson employed long sequences composed of 20 elements. The randomness of a sequence may be hard to perceive directly with such a long sequence. These considerations are in line with the idea that the processes underlying the classification task within randomness perception may be sensitive to many factors: task instructions, the length of the sequence to be judged, and even, all things being equal, the mindset of the participant that will determine which one of the two strategies (or even both) will be employed.

Future research should also provide a complete description of the time course of information processing of the mechanisms involved in both single-sequence tasks (common in the randomness perception literature) and in comparison tasks that involve two stimuli (as used here and in Zhao et al., 2014). This requires identifying both the stopping rule used to make a decision, and the degree of independence of the subprocesses. The two subprocesses can take place one at time (serial processing) or at the same time (parallel processing). The system will await the completion of both subprocesses (exhaustive processing) or it will stop as soon as the first subprocess is completed (minimum time stopping rule). In the case of probabilistic processing, are the two subprocesses stochastically independent? In the case of two stimulus strings, is there a serial, privileged order-of-analysis or is the analysis of both sequences in parallel? Answering these and other questions (such as the capacity of the channels) is necessary to understand how the two subprocesses are organized in randomness perception.

Reviewing the literature about reaction times and serial/parallel processing, Algom et al. (2015) indicate that the double factorial paradigm (DFP; Eidels et al., 2010; Townsend & Nozawa, 1995) is the current gold standard for distinguishing different models obtained from the combination of features listed above. DFP requires two simultaneous manipulations across the two subprocesses, each producing a factorial design: The manipulation of the workload of the system (e.g., varying the number of targets) and the manipulation of saliency of stimulus features (e.g., varying the readability of the stimulus’ font). The application of DFP may be straightforward in the domain of visual search and similar domains. However, its application to randomness perception is difficult because it requires manipulating the difficulty of encoding strings and their representativeness independently. This is nontrivial because the two properties are strongly correlated. Possible manipulation that could help may be employing sequences of different lengths, using fonts with different readability (XO vs. YV), manipulating the space between the characters of the sequence, or using different kind of instruction explicitly worded (look for any regularities vs. find the sequence that is more similar to reference stimulus).

In this study we manipulated the instructions across two independent groups of subjects: Future studies should verify if the effect is present in a within-subjects design in order to obtain a clearer picture.

The ambiguity of the term “randomness” and related concepts (Falk, 1991; Gnedenko, 1962; Nickerson, 1996) makes the study of randomness perception tricky. Tasks are often ill-defined and participants’ understanding of them is not straightforward (Nickerson, 2002). The hypothesis of a mixed strategy comprising both encoding and representativeness fits with the idea that “instructions can bias participants to look for specific characteristics of sequences as evidence of randomness, or they can be sufficiently vague to permit more than one interpretation. . . . It is important to consider how participants understood the task, and it is not safe to assume that they invariably did so as the experiment intended” (Nickerson, 2002, p. 351).

Given the common tacit assumption that the two possible answers to a single question are determined by the same cognitive processes, our observations are generally compatible with the previous literature (Falk & Konold, 1997; Oskarsson et al., 2009; Reimers et al., 2018), where results can be explained both in terms of similarity-based judgment with a prototype or an active search for possible regularities.

More recent studies (Hahn & Warren, 2009; Reimers et al., 2018; Sun & Wang, 2010) of the perception of randomness have focused on the comparison between the heuristics and biases account with an explanation based on rational judgment characterized by limited experience (i.e., an adaptive response to real-world phenomena showing an excess of alternation). Comparing these two explanations, Reimers et al. (2018) found evidences for the biased judgement account and that people use heuristics based on several distinct forms of representativeness. The authors include in the representativeness heuristic an evaluation of the complexity of the sequence (what we have called the encoding strategy) because sequences highly representative of a random process are hard to chunk. They observed that strategies include the evaluation of the alternation rate, the relative proportions of the outcomes and the difficulty encoding of the sequence (Reimers et al., 2018).

Miller and Sanjurjo (2018) and Sun et al. (2015) have questioned the idea that the randomness perception of people is actually biased: indeed, the overalternating bias may be consistent with the statistical properties of sequential data in natural environments. Our findings are not incompatible with this perspective in that we focused on the particular strategy (similarity criterion or regularity finding) that people adopt in a randomness judgment, regardless of whether such decisions satisfy a rational criterion or not.

Understanding whether an event is random or not is a central topic in many everyday situations and research activities. This work suggests that understanding randomness may rely on a similarity criterion, an attempt to look for regularities, or both. When judging an event, an individual may try to see if it resembles the mental prototype of a random event, or try to look for regularities, or employ a mixed strategy that does both. This insight should help us understand phenomena like superstitious thinking, when and why people find structure in sequences, even when it is not there.

## Supplementary Information

ESM 1 (DOCX 1481 kb)

## Footnotes

1 The DP measure, although very successful in modelling subjective randomness judgments, quantifies the randomness of a string only in terms of the number of repeating and alternating elements. However, other attributes of sequence of elements (such as symmetry, complementarity, and so on) may determine the subjective randomness of a string. Griffiths and Tenenbaum (2003, 2004; Griffiths et al., 2018) developed a Bayesian model of randomness judgments able to grasp even these attributes (and their relative weight in the final judgement). Given that there is a very high correlation between their measure and DP (*r* = .94; Gronchi et al., 2016) when computed on the set of 128 strings employed in the experiment, we employed DP as a measure of subjective randomness.

2 Analyzing the subset of strings with peculiar attributes (symmetry, complementarity, and so on, as described by Griffiths & Tenenbaum, 2003, 2004) we observed that these sequences are associated with faster reaction times, but the overall results do not change.

3 As Kubovy and Gilden (1991) noted, Kahneman and Tversky (1972) already showed that this hypothesis is partially correct. According to them, the lack of order is not a sufficient condition to affirm that the string is random; it is necessary that it is representative of random sequences.
