# Supplementary material for: Regular and random judgements are not two sides of the same coin: Both representativeness and encoding play a role in randomness perception
Source: Psychon Bull Rev. 2021 May 6;28(5):1707–14. doi: 10.3758/s13423-021-01934-9 (PMC8500893; doi:10.3758/s13423-021-01934-9)

1) Violin plot with box-plot of Log_10_ reaction times as function of the complexity (low/intermediate, low/high and intermediate/high) of pairs of sequences, condition (selecting the sequence generated by the random source vs selecting the sequence generated by the Nonrandom source) and the complexity of the leftmost sequence


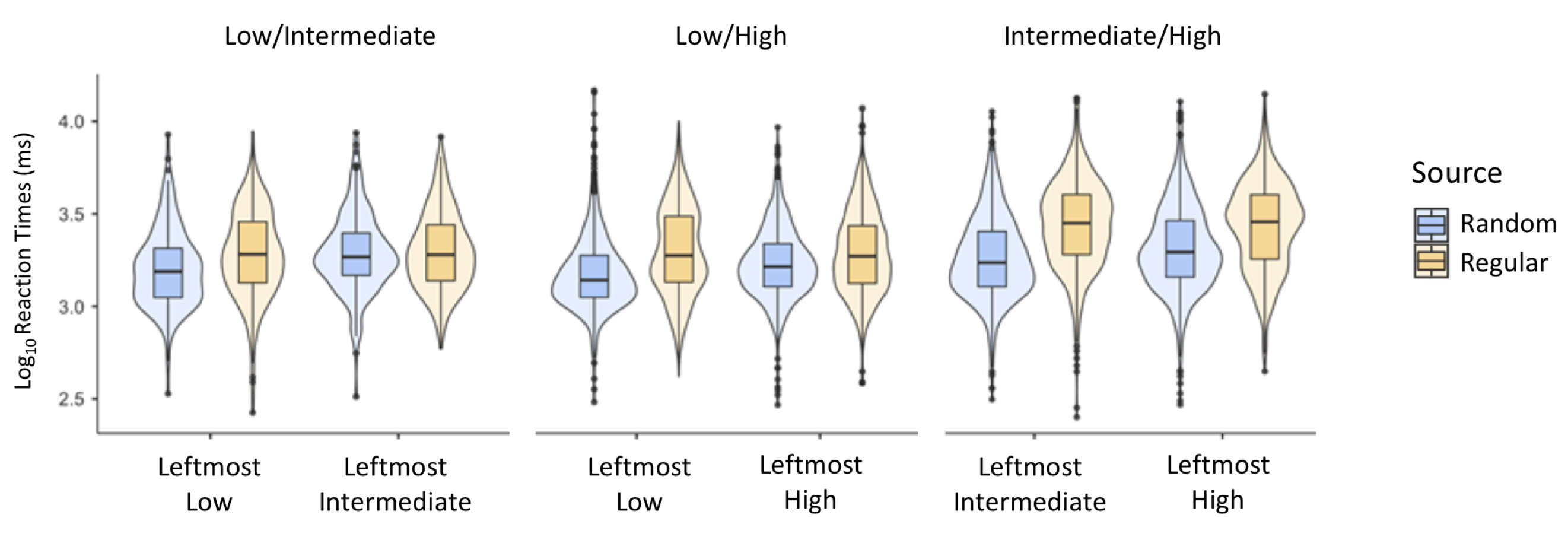


2) Violin plot with box-plot of Log_10_ reaction times as function of the complexity (low/low, intermediate/intermediate and high/high) of pairs of sequences, condition (selecting the sequence generated by the random source vs selecting the sequence generated by the Nonrandom source) and the selected string (left or right).


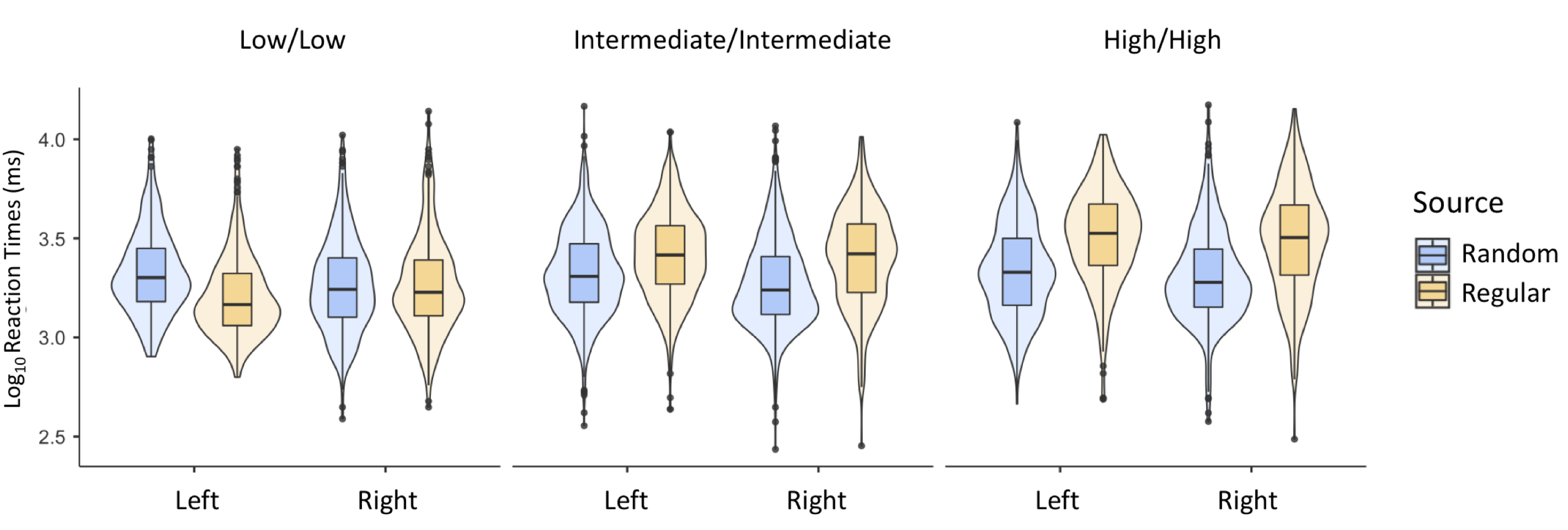


3) Violin plot with box-plot of Log_10_ reaction times as function of the complexity (low/intermediate, low/high and intermediate/high) of pairs of sequences, condition (selecting the sequence generated by the random source vs selecting the sequence generated by the Nonrandom source) and the complexity of selected string.


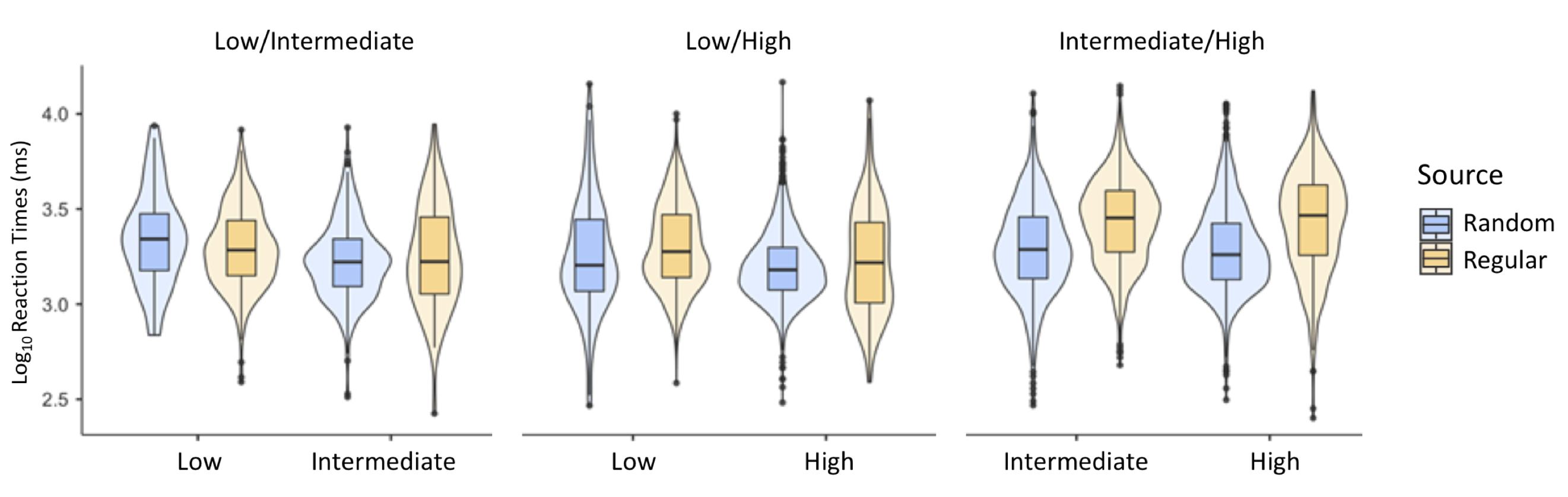

Supplement: Supplementary file 1 — (DOCX 1481 kb) [file 13423_2021_1934_MOESM1_ESM.docx]
